# Structure of *Plasmodium falciparum* orotate phosphoribosyltransferase with autologous inhibitory protein–protein interactions

**DOI:** 10.1107/S2053230X1500549X

**Published:** 2015-04-21

**Authors:** Shiva Kumar, Kalyanaraman Krishnamoorthy, Devaraja G. Mudeppa, Pradipsinh K. Rathod

**Affiliations:** a Department of Chemistry, University of Washington, Seattle, WA 98195, USA

**Keywords:** pyrimidine, *de novo*, salvage, PRPP, OMP, prodrugs, peptide inhibition

## Abstract

The most severe form of malaria is caused by the obligate parasite *Plasmodium falciparum*. Orotate phosphoribosyltransferase (OPRTase) is the fifth enzyme in the *de novo* pyrimidine-synthesis pathway in the parasite, which lacks salvage pathways. Among all of the malaria *de novo* pyrimidine-biosynthesis enzymes, the structure of *P. falciparum* OPRTase (*Pf*OPRTase) was the only one unavailable until now. *Pf*OPRTase that could be crystallized was obtained after some low-complexity sequences were removed. Four catalytic dimers were seen in the asymmetic unit (a total of eight polypeptides). In addition to revealing unique amino acids in the *Pf*OPRTase active sites, asymmetric dimers in the larger structure pointed to novel parasite-specific protein–protein interactions that occlude the catalytic active sites. The latter could potentially modulate *Pf*OPRTase activity in parasites and possibly provide new insights for blocking *Pf*OPRTase functions.

## Abstract 2

*P. falciparum* orotate phosphoribosyltransferase, a potential target for antimalarial drugs and a conduit for prodrugs, crystallized as a structure with eight molecules per asymmetric unit that included some unique parasite-specific auto-inhibitory interactions between catalytic dimers.

## 1. Introduction   

Historically, malaria has caused sustained morbidity and mortality in the human population. Many believe that the disease has imposed the most potent selective pressure in the recent evolution of the human genome (Kwiatkowski, 2005 ▶). The problem continues. In the last 20 years, malaria-related deaths increased from <1 million to 1.8 million in 2004 before decreasing to the current estimate of about 600 000 per year (Murray *et al.*, 2012 ▶). As encouraging as the recent numbers are, sustained management of malaria morbidity and mortality will require new treatments, including new drug targets (Dhanawat *et al.*, 2009 ▶; Gamo *et al.*, 2010 ▶).

The most severe form of malaria is caused by the obligate parasite *Plasmodium falciparum*, particularly in Africa. These intracellular parasites can be selectively attacked by inhibitors of essential enzymes. Potent and selective antimalarials have been developed to target *P. falciparum* dihydroorotate dehydrogenase, the fourth enzyme in the *de novo* pyrimidine-biosynthesis pathway (Baldwin *et al.*, 2005 ▶; Phillips *et al.*, 2008 ▶; Coteron *et al.*, 2011 ▶; Deng *et al.*, 2014 ▶; Gujjar *et al.*, 2011 ▶). Recently, one triazolopyrimidine derivative from this initiative (DSM265) has advanced to early human testing (http://www.mmv.org/research-development/rd-portfolio).

**Figure 1:**
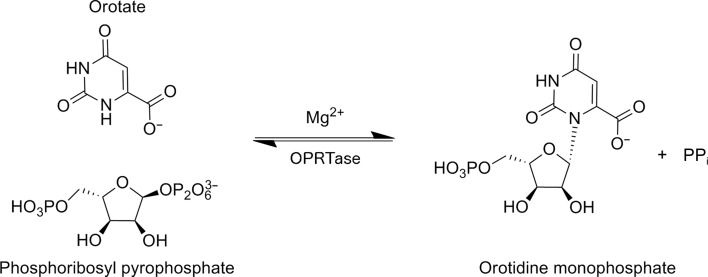
The orotate phosphoribosyltransferase (OPRTase) reaction. OPRTases catalyze the conversion of orotate and 5-phospho-d-ribosyl 1-pyrophosphate (PRPP) to orotidine 5′-monophosphate (OMP).

The pipeline for antimalarials could benefit from additional good targets and strategies that revolve around *de novo* pyrimidine metabolism in the parasite. Specifically, *P. falciparum* orotate phosphoribosyltransferase (*Pf*OPRTase), the fifth enzyme in the indispensible *de novo* pyrimidine-synthesis pathway of the parasite (Reyes *et al.*, 1982 ▶), is of special interest. OPRTase catalyses the conversion of orotate and 5-phospho-d-ribosyl 1-pyrophosphate (PRPP) into orotidine 5′-monophosphate (OMP) (Fig. 1 ▶). While OPRTase and OMP decarboxylase, the next enzyme in the *de novo* pyrimidine-synthesis pathway, are fused into one bifunctional enzyme in humans, they exist as two different gene products in the malarial parasite (Rathod & Reyes, 1983 ▶). OPRTase is a dimeric enzyme with two active-site pockets, one on each protomer. Once the substrate has docked, two different loops, one from each protomer, cover the bound substrate to shield it from solvent (Scapin *et al.*, 1994 ▶; Henriksen *et al.*, 1996 ▶). The OPRTase protein sequence is 100% identical between the two strains of *P. falciparum*, 3D7 and IT, as seen in PlasmoDB (Aurrecoechea *et al.*, 2009 ▶). Additionally, among the 203 single-nucleotide polymorphism (SNP) data sets in Plasmo­DB, *Pf*OPRTase contains 30 SNPs. Two of these are stop-codon SNPs, 25 are nonsynonymous and three are synonymous. Previously, *Pf*OPRTase has been used as a conduit for potent and selective prodrugs directed at downstream enzymes in pyrimidine biosynthesis (Rathod *et al.*, 1989 ▶; Gómez & Rathod, 1990 ▶). Potent direct inhibitors of *Pf*OPRTase remain to be discovered.

The availability of an atomic resolution structure of *Pf*OPRTase could help to reveal the precise layout of the substrate and prodrug-binding pocket, which in turn could help with the design of selective inhibitors. Unfortunately, like many other important malarial proteins, *Pf*OPRTase has eluded structure determination. Molecular features unique to *Plasmodium* genes and gene products, such as AT-rich tracks, polyasparagine repeats and the binding of protein by cognate mRNA (Zhang & Rathod, 2002 ▶), can interfere with the heterologous expression of malaria proteins (Schneider *et al.*, 2005 ▶) and can also promote protein aggregation (Singh *et al.*, 2004 ▶). This has resulted in an underrepresentation of structures from *Plasmodium* proteins in the Protein Data Bank (wwPDB) that is even worse than for that of membrane proteins (Vedadi *et al.*, 2007 ▶; Mehlin *et al.*, 2006 ▶).

In 2012, a preliminary publication reported the crystallization of *Pf*OPRTase (Takashima *et al.*, 2012 ▶). However, to date there is no public information on the underlying quality of the data from this crystal or an OPRTase structure. Here, we report the optimum expression and crystallization of *Pf*OPRTase. Our structure reveals novel parasite-specific protein–protein interactions that are of possible importance in malaria biology. In addition, we can map the positions of active-site amino-acid residues that differ between the host and the parasite OPRTases and this should facilitate future new structure-based drug-development strategies.

## 2. Materials and methods   

### 2.1. Protein production   

**Figure 2:**
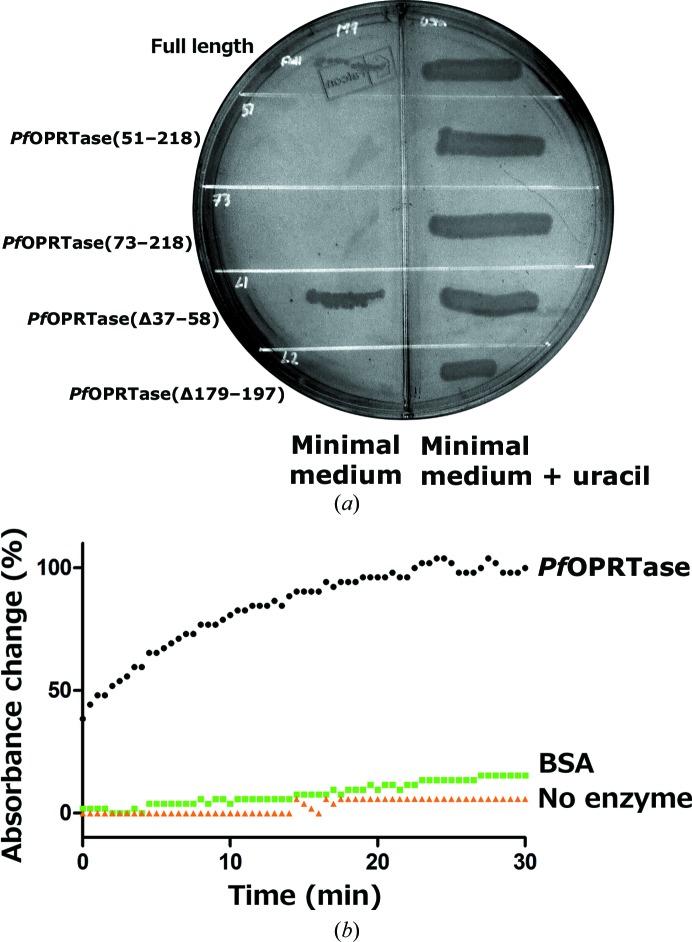
Activity assays validating functional *Pf*OPRTase constructs. (*a*) Select *Pf*OPRTase constructs can support the growth of OPRTase-deficient *E. coli*. Several variants of *Pf*OPRTase-coding sequences [full length, *Pf*OPRTase(51–218), *Pf*OPRTase(73–218), *Pf*OPRTase(Δ37–58) and *Pf*OPRTase(Δ179–197)] in plasmid pET-28a were transformed into *E. coli* JW3617 cells. The cells were grown in M9 minimal medium (left half of the plate) or M9 medium supplemented with uracil (right half of the plate). Full-length *Pf*OPRTase and partially deleted *Pf*OPRTase (Δ37–58) successfully rescued *E. coli* JW3617 cells from OPRTase deficiency, but not the others. (*b*) Time-dependent reduction in absorbance as orotic acid is converted to orotidylate (OMP) by functional *Pf*OPRTase. The change in the absorbance by orotic acid (at 296 nm) is shown in the presence of *Pf*OPRTase (black), bovine serum albumin (green) or no added enzyme (orange).

Conventional methods for heterologous protein overexpression were not useful for producing *Pf*OPRTase in *Escherichia coli* in the quantities required for screening crystallization conditions. Codon optimization to compensate for the unusual AT-rich *Plasmodium* genes failed to give desirable yields in *E. coli*. The expression of functional *Pf*OPRTase was aided by obligatory protein production in auxotrophic bacteria, as previously performed to obtain *P. falciparum* serine hydroxymethyltransferase (Alfadhli & Rathod, 2000 ▶). Bacterial strain JW3617(DE3) from the *E. coli* stock centre (Yale University) was grown in M9 minimal medium (Sigma, St Louis, Missouri, USA) with *Pf*OPRTase function provided on a pET-28a vector (Merck KGaA, Darmstadt, Germany). Since the JW3617(DE3) cells lacked a native OPRTase gene, their survival depended on retaining a functional *Pf*OPRTase. This system allowed us to explore different constructs of *Pf*OPRTase for improved yield while retaining function. We hypothesized that diffraction-quality crystals may be best obtained by reducing the packing disorder caused by surface entropy. Various lengths of the polyasparagine repeats, which are unique to *P. falciparum* and do not appear to contribute to catalytic function, were individually deleted. In addition, a series of N-terminal truncations were also tested. The ability to rescue OPRTase function in JW3617 (DE3) cells in M9 minimal medium agar was used to identify functionally active constructs. Using this criterion, in addition to full-length *Pf*OPRTase-6×His, *Pf*OPRTase 1–218(Δ37–58)-H6 with a C-terminal six-His tag appeared to be promising (Fig. 2 ▶ *a*).

*Pf*OPRTase protein was expressed in JW3617(DE3) cells grown in M9 medium supplemented with 40 µg ml^−1^ kanamycin. The cells were grown at 310 K until the OD_600_ reached 0.7–0.8. Subsequently, the cells were transferred to 291 K and, after 30 min, *Pf*OPRTase production was induced with 100 µ*M* IPTG. The cells were harvested 16 h after induction for protein extraction and purification. *Pf*OPRTase 1–218(Δ37–58)-H6 (or full-length *Pf*OPRTase-6×His) was released by lysing the host *E. coli* cells in buffer *A* (50 m*M* Tris–HCl pH 7.3, 150 m*M* NaCl, 25 m*M* imidazole, 5% glycerol, 2 m*M* β-mercapto­ethanol) using a sonicator. The lysate was clarified by centrifugation (18 000*g*, 277 K, 20 min). The protein supernatant was passed through a 5 ml HisTrap FF column (GE Healthcare Biosciences, Pittsburgh, Pennsylvania, USA), after which the column was washed with ten volumes of buffer *A*. Adsorbed protein was then eluted off the column by passing five column volumes of elution buffer (buffer *A* with 250 m*M* imidazole–HCl). An Amicon concentrator (Merck KGaA, Darmstadt, Germany) was used to equilibrate the protein with low-salt buffer *B* (50 m*M* Tris–HCl pH 7.3, 50 m*M* NaCl, 5% glycerol, 2 m*M* β-mercapto­ethanol) before anion-exchange chromatography. The protein in buffer *B* was then loaded onto a 5 ml HiTrap Q-Sepharose FF column (GE Healthcare) and purified using a gradient from buffer *B* to buffer *C* (50 m*M* Tris–HCl pH 7.3, 1000 m*M* NaCl, 5% glycerol, 2 m*M* β-mercapto­ethanol). The eluted protein was then injected onto an S200 XK16/60 size-exclusion chromatography column (GE Healthcare Biosciences) for final purification. The column was run on an ÄKTA FPLC system (GE Healthcare Biosciences) using buffer *D* (20 m*M* Tris–HCl pH 7.3, 100 m*M* NaCl, 0.5 m*M* TCEP). The final protein was concentrated to 30 mg ml^−1^ before crystallization.

### 2.2. *In vitro* catalytic activity of purified protein   

The final purified protein was tested for catalytic activity before use in crystallization experiments. Purified *Pf*OPRTase 1–218(Δ37–58)-H6 (50 n*M*) was mixed with the substrates phosphoribosyl pyrophosphate (2 m*M*) and orotic acid (100 µ*M*) in a buffer consisting of 50 m*M* Tris–HCl pH 7.5, 150 m*M* NaCl, 2 m*M* MgCl_2_, 1 m*M* TCEP. The decrease in absorbance was measured at 296 nm (Mudeppa & Rathod, 2013 ▶). Orotic acid utilization was observed with purified *Pf*OPRTase, but not with BSA or with no protein added (Fig. 2 ▶ *b*). The catalytic activity confirmed that the heterologous *Pf*OPRTase 1–218(Δ37–58)-H6 protein was purified in an active form.

### 2.3. Protein crystallization   

The protein was concentrated to 30 mg ml^−1^ before crystallization. Full-length *Pf*OPRTase-6×His did not crystallize under the conditions tested, but *Pf*OPRTase 1–218(Δ37–58)-H6 did. Diffraction-quality crystals were grown in hanging-drop plates (Hampton Research) in 100 m*M* ammonium sulfate, 30% PEG 3350 at 277 K. Crystals were cooled in liquid nitrogen using the reservoir solution supplemented with 20% ethylene glycol. Diffraction data were collected on beamline 23-ID-B at the Advanced Photon Source (APS), Argonne National Laboratories, Argonne, Illinois, USA.

### 2.4. Structure determination   

*Pf*OPRTase 1–218(Δ37–58)-H6 crystallized in the primitive orthorhombic space group *P*2_1_2_1_2_1_, with unit-cell parameters *a* = 114.769, *b* = 152.487, *c* = 167.755 Å (Table 1 ▶). Estimating the precise space group was not trivial at the beginning of structure determination. The unit-cell volume was very large for a 31.37 kDa protein. Based on the Matthews coefficient, we expected eight to ten molecules in the asymmetric unit (Matthews, 1968 ▶; Kantardjieff & Rupp, 2003 ▶). The potentially large number of expected protein molecules in the asymmetric unit (*n*_mol_) made it difficult to conclusively predict their exact number *a priori*. We arrived at *n*_mol_ = 8 by successively placing the molecules correctly in the asymmetric unit. A high number of molecules in an asymmetric unit can sometimes lead to a noncrystallographic symmetry axis within the asymmetric unit that is parallel to a crystallographic symmetric axis. The resulting possibility of pseudo-translation was tested by drawing a native Patterson map, and this gave a single off-origin peak of height 46% at fractional coordinates 0, 0.5, 0.5. Given that the vector, at a distance of 113 Å from the origin, was 46% of the origin, it was concluded that it was not a crystallographic *A*-centering vector but a noncrystallographic symmetry vector. Systematic absences of a 2_1_ screw operator on either *b*, *k* = 2*n*, or on *c*, *l* = 2*n*, can satisfy the condition for systematic absences for *A*-centering, with *k* and *l* both being odd or even. This made it hard to discern whether a 2_1_ screw was indeed present in the crystal at the *b* axis, at the *c* axis or both. In addition, molecular-replacement searches were expected to give a very low signal-to-noise ratio owing to the small scattering volume of the search model in relation to the asymmetric unit. Furthermore, the best molecular-replacement (MR) search model, *Saccharomyces cerevisiae* OPRT (PDB entry 2pry; González-Segura *et al.*, 2007 ▶), had 33% sequence identity, which by itself could reduce the signal-to-noise ratio for the MR searches. A mammalian OPRTase domain from PDB entry 2wns (Structural Genomics Consortium, unpublished work) had a sequence identity of 24% using a *BLAST* alignment, which made the *S. cerevisiae* structure best suited as an MR search model.

Nevertheless, MR searches were carried out in all of the possible space groups of the point group *P*222. Using data processed and scaled in *HKL*-2000 (Otwinowski & Minor, 1997 ▶), initial MR searches in *MOLREP* (Vagin & Teplyakov, 2010 ▶), *AMoRe* (Navaza, 1994 ▶) and *Phaser* (McCoy *et al.*, 2007 ▶) as part of the *CCP*4 suite (v.6.3.0) failed to provide any solution with good signal when compared with a random position in the asymmetric unit. Next, all of the solutions provided by *Phaser* (v.2.5.2) while searching for the first ensemble were visually inspected in *PyMOL* (v.1.5.0.4; Schrödinger). Two solutions which looked like possible catalytic dimers were selected and fixed. The search was then repeated to find further solutions. The signal-to-noise ratio in the *Phaser* output improved as more molecules were chosen manually by visual inspection of pairs of polypeptides that seemingly formed a catalytic dimer. They were fixed in the asymmetric unit. Further solutions were automatically found by *Phaser*. After finding all eight molecules, *phenix.autobuild* (Adams *et al.*, 2010 ▶) was used to construct the initial structure. Further building was performed manually using *Coot* (Emsley *et al.*, 2010 ▶), while iterative refinement was performed using *REFMAC* (Murshudov *et al.*, 2011 ▶).

## 3. Results   

### 3.1. Crystal packing   

**Figure 3:**
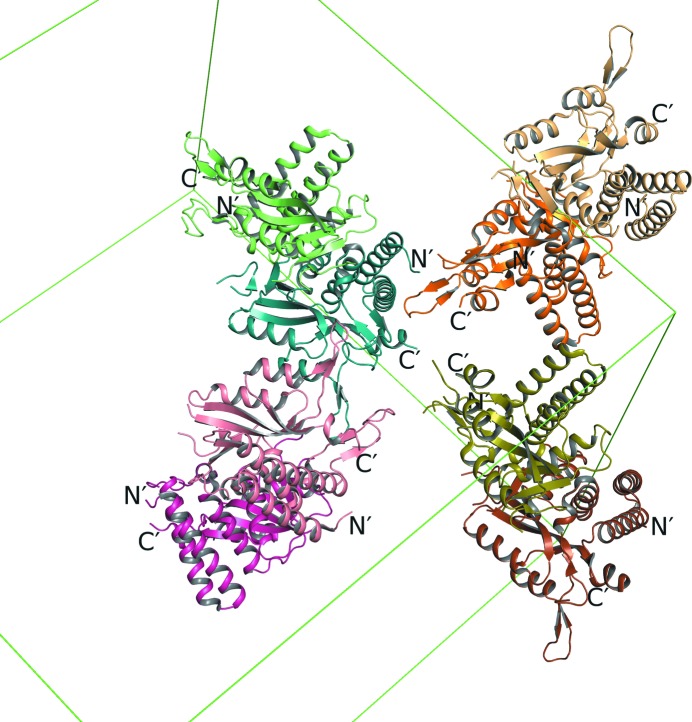
*P. falciparum* OPRTase crystal packing. *Pf*OPRTase packs with eight molecules in the asymmetric unit. All of the chains are shown in different colors: chains *A* and *B* in light orange and dark orange, chains *C* and *D* in light green and blue-green, chains *E* and *H* in light pink and dark pink and chains *F* and *H* in olive and brown, respectively. Catalytic dimers are colored in different shades of the same color. The unit cell is shown with green lines. The N- and C-termini of all chains are labeled. The rotation axis of the catalytic dimer is nearly parallel to the crystallographic axis, which caused the observed pseudo-translation.

In our crystal, *Pf*OPRTase 1–218(Δ37–58)-H6 packs as a set of four catalytic dimers, leading to eight molecules in the asymmetric unit (Fig. 3 ▶). None of the protomers in the four dimers are related by a crystallographic twofold. Instead, they are related by a near-perfect noncrystallographic twofold rotation (denoted here as κ) with κ ranging from 179.3 to 179.8°.

### 3.2. Two sites in an obligatory dimer   

**Figure 4:**
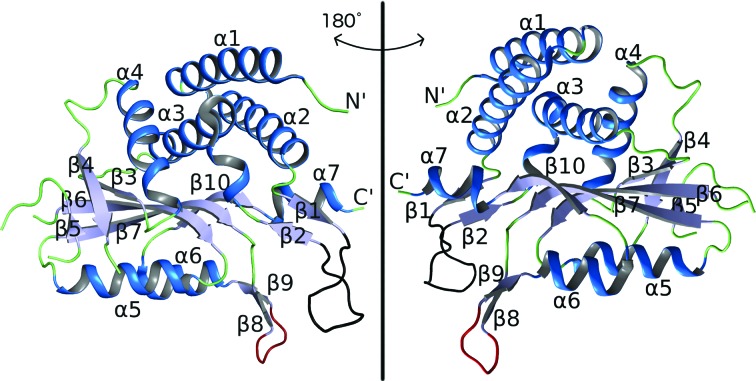
Structure of an individual subunit of *Pf*OPRTase. The two views depict a 180° horizontal rotation. All of the helices and strands are numbered according to their order of occurrence from the N-terminus of the protein subunit. Helices are shown in dark blue, β-strands in light blue and most loops in green. The loop in red between β8 and β9 (^247^NENNQ^251^) makes interdimer contacts (see §[Sec sec3]3 and Fig. 7 ▶). The loop in black between β1 and β2 (^84^GEFILKSKRKSN^95^) forms a ‘hood’ that is expected to wrap around the substrates (see §[Sec sec3]3 and Fig. 6 ▶)

In the present crystal, the individual *Pf*OPRTase molecules possess the conserved α/β topology also seen in other OPRTases (Scapin *et al.*, 1994 ▶; Henriksen *et al.*, 1996 ▶; Liu *et al.*, 2010 ▶). There are seven α-helices and ten β-strands in the structure, in addition to one *cis*-peptide (Fig. 4 ▶). The *Pf*OPRTase structure conforms with the conserved PRTase fold, having a 321456 arrangement of β-sheets. The antiparallel strand 3 of this sheet is not tightly ordered into a distinctive β-strand in the solved structure.

**Figure 5:**
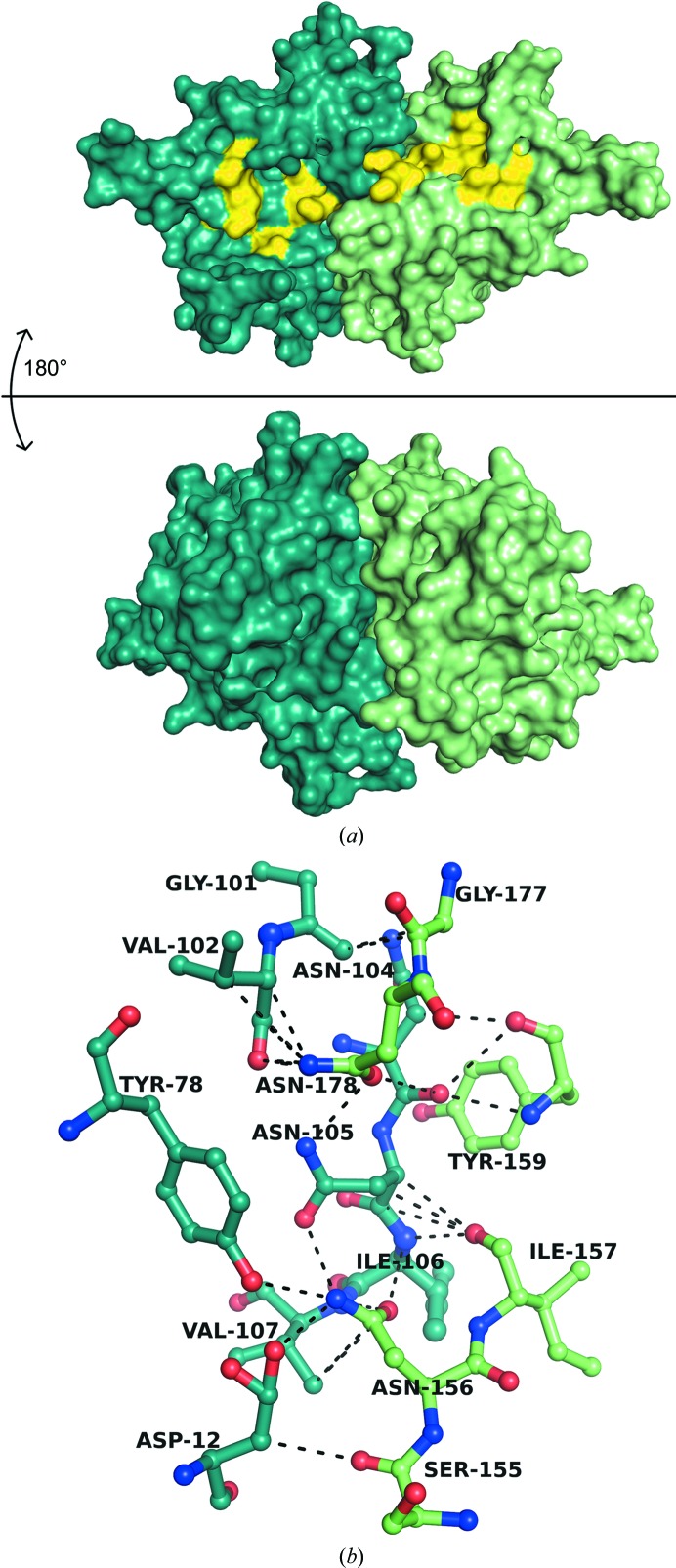
Description of the OPRTase obligate dimer. (*a*) Two subunits contribute to each of the two active sites of the *Pf*OPRTase dimer. The two views, 180° apart, represent the surfaces of the two protomers (colored blue and green) of the *Pf*OPRTase dimer with a large buried surface area of 1500 Å. The substrate-binding site on one face of the dimer is colored yellow. (*b*) The catalytic dimer of *Pf*OPRTase is held together primarily by interprotomer hydrogen bonds, with contributions from one protomer shown in dark green and those from the other in light green.

At the core, the *Pf*OPRTase 1–218(Δ37–58)-H6 molecules pack as dimers. This is consistent with OPRTase structures from other organisms, which have shown that a dimer is essential to form catalytic active sites (Scapin *et al.*, 1994 ▶; Henriksen *et al.*, 1996 ▶; Liu *et al.*, 2010 ▶). The catalytic dimers have an average buried surface area of 1500 Å^2^ (Fig. 5 ▶ *a*). Among the database of OPRTase structures from other source organisms in the wwPDB, *Pf*OPRTase is the most closely related to that from *S. cerevisiae* (*Sc*OPRTase) in sequence as well as in structure. *Pf*OPRTase differs from *Sc*OPRTase with an r.m.s.d. of 2.1 Å (calculated by SSM Superpose in *Coot*; Emsley *et al.*, 2010 ▶), whereas with the human enzyme (PDB entry 2wns) the value is 2.6 Å. The interfaces between all of the four catalytic dimers in the *Pf*OPRTase asymmetric unit are identical. The interface between chains *C* and *D* (PDB entry 4fym) is used in the present description since it has the best electron density of the four dimers. The catalytic dimer in our *Pf*OPRTase structure is held together exclusively by hydrogen bonds (Fig. 5 ▶ *b*). In particular, α4 and the loop residues leading up to it make dominant interactions that hold the catalytic homodimer together. Almost all of the surface residues of α4, which is comprised of the residues ^136^KGIPMVSLTSHFLFE^150^, are buried at this dimer interface. The α4 helix then leads to β4, which also makes predominant contacts at the interface.

### 3.3. Substrate-binding sites   

Each of the two active sites of *Pf*OPRTase receives amino-acid contributions from the two polypeptides of the dimer. Beyond the dimer-interacting amino acids discussed above, there are eight disordered amino acids in the *Pf*OPRTase structure. These eight amino acids have previously been observed to be disordered in the apo form of the yeast enzyme (PDB entry 2ps1; González-Segura *et al.*, 2007 ▶). These eight disordered amino acids (^165^EKKE­YGDK^172^ from each sub­unit of the dimer in *Pf*OPRTase) are expected to wrap around PRPP based on available structures with substrate. PRPP is also bound to the dimeric partner, and together the two polypeptides contribute to the catalytic reaction (Henriksen *et al.*, 1996 ▶). This stretch of eight amino acids leads into β5, which forms the only antiparallel strand of the conserved OPRTase β-sheet. Gly84 and Gly101 play an important role as hinge residues to provide flexibility to the hood domain. The hood domain moves on top of the substrate to sequester it from free solvent. The hood domain contains Phe97, which is known to form a π-stacking interaction with the substrate orotate (González-Segura *et al.*, 2007 ▶). Gly101 and Val102 at one hinge of the hood are part of a 3_10_-helix and they interact with β5 at the dimeric interface. This interaction holds the hood domain in the correct orientation to make the π-stacking possible.

The sulfates in the *Pf*OPRTase structure provide excellent clues to the detailed positioning of the natural phosphate-containing substrates and products of *Pf*OPRTase. There are 2–6 sulfate ions originating from the crystallization conditions associated with each polypeptide in the core dimer. They occupy the same positions in each of the eight chains in the asymmetric unit. The exact number of sulfates varies between the eight *Pf*OPRTase chains owing to differences in the quality of the electron density in the individual polypeptides. The two most interesting sulfate ions occupy the positions where the phosphate and pyrophosphate groups of the PRPP substrate are expected to bind. From the homologous structures of yeast OPRTase bound to PRPP (PDB entries 2ps1 and 1lh0; González-Segura *et al.*, 2007 ▶; Grubmeyer *et al.*, 2012 ▶), we can predict where the PRPP phosphates would make contact with the *Pf*OPRTase polypeptide. The 5′-phosphate should make contacts with the pocket formed by ^212^TCGTA^216^ (Supplementary Fig. S1), while the terminal phosphate of the 1′-pyrophosphate is expected to contact the scaffold formed by ^135^YK^136^. In addition to the active-site sulfates, a sulfate close to the N-terminus mediates some of the contacts between α1 of one protomer and the loop between α4 and β4 of the other protomer. Three water molecules that coordinate this sulfate ion also help to mediate interactions between the two protomers. Two other sulfates are present at the interface between α4 of the two protomers of each dimer. Residues from the two protomers coordinate each of these two sulfates.

**Figure 6:**
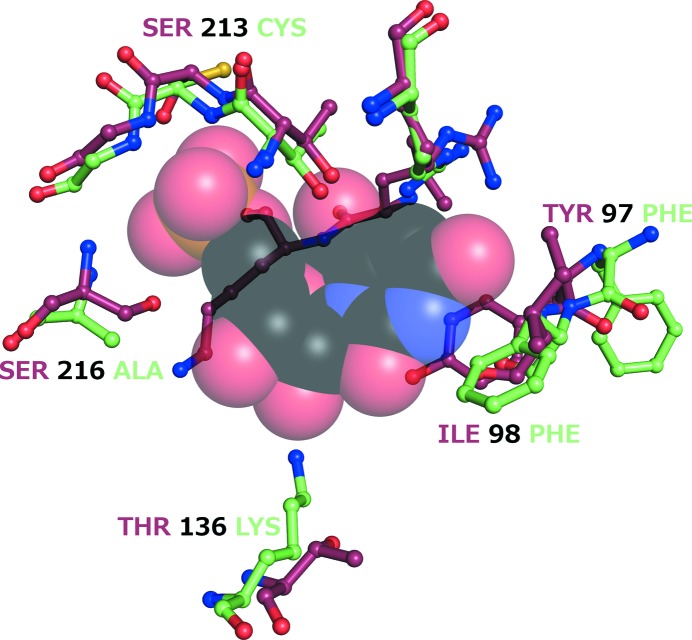
Host–parasite differences in the active site of OPRTase. Differences in key amino acids are color-coded, with the human enzyme structure in green and the malaria parasite structure in red. The space-filling model depicts the position of the product orotidylate (OMP) from the human OPRTase structure. The amino acids in green are those found within 4 Å of OMP (see §[Sec sec3.3]3.3).

A comparison of host–parasite differences in the OPRTase active-site residues within 4 Å of OMP in the mammalian structure (PDB entry 2wns) points to several differences that could contribute to the development of specific antimalarial inhibitors. Fig. 6 ▶ shows that Lys136 in the parasite enzyme, which interacts with an active-site sulfate ion that is thought to interact with the 1′-phosphate of PRPP, is a Thr in humans. In addition, Cys213 and Ala216 in the parasite enzyme replace the two Ser side chains in the human enzyme that normally interact with the 5′-phosphate of OMP. Phe97 in the malarial enzyme replaces the Tyr residue of the human enzyme known to form a stacking interaction and a hydrogen bond with orotate. A neighboring Phe98 in *Pf*OPRTase replaces an Ile which forms main-chain interactions with OMP in the human enzyme. Together, these mutations provide hope that it should be possible to find low-molecular-weight inhibitors that bind the parasite enzyme with selectivity.

### 3.4. Parasite-specific inhibitory protein–protein interactions   

The individual repeat dimers within the *Pf*OPRTase crystal form unexpected malaria-specific quaternary arrangements that have not been reported for other OPRTases. Among the four catalytic dimers in the asymmetric unit, two are related by an additional noncrystallographic symmetry: a dimer of catalytic dimers form an almost perfect twofold symmetry with κ = 179.97°.

**Figure 7:**
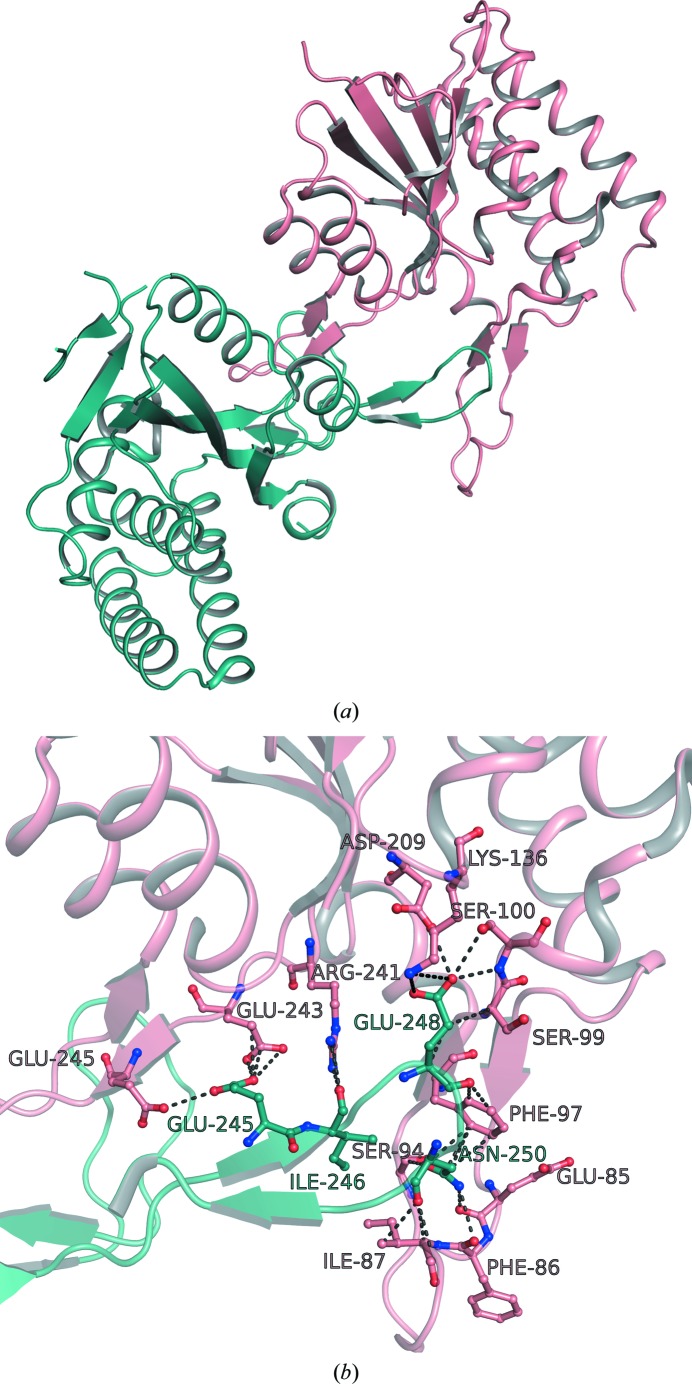
Intersubunit inhibitory interactions. (*a*) Unique, parasite-specific, interdimer contacts involving the potentially inhibitory insertion of a loop from one dimer (green) and the active site of an adjacent dimer (pink). (*b*) Details of the interdimer contacts showing hydrogen-bond inter­actions that stabilize the incoming loop (green) and that help to form a defined structure on the open ‘hood’ (Glu85, Phe86 and Ile87).

In this larger tetrameric structure, a protomer from one catalytic dimer forms an inhibitory interaction with the active site of another protomer but from a different neighboring catalytic dimer (Fig. 7 ▶). A β-hairpin formed by β8 and β9 (^243^EYEINENNQKIY^254^; Supplementary Fig. S2) inserts itself exactly at the substrate-binding pocket of the recipient active site. This completely occludes the closure of the hood domain and would prevent crucial substrate interactions, including π-stacking with Phe97, in the hood domain. The buried surface area at this inhibitory interface is ∼1170 Å^2^. Twofold symmetry at this inhibitory interface leads to completely isologous interactions. The same residues are involved in the same type of interactions on each of the monomer pairs, not within a dimer but between dimers. The interface hotspot is at Glu248, which makes a salt bridge with Lys136 on the other protomer. Glu248, which is part of the β-hairpin, is buried at the inhibitory interface and forms additional hydrogen bonds to Ser100 and Asp209. Another residue of the β-hairpin, Asn250, also makes crucial inter­actions (Fig. 7 ▶). It acts as a fulcrum around which the hood domain of its dimeric partner wraps itself, making extensive hydrogen bonds *via* the main-chain atoms of Glu85, Phe86 and Leu88 and the side-chain atoms of Ser94. Presumably, these intersubunit interactions blocking the active site of *Pf*OPRTase occurred at high crystallization concentrations, but they hint at protein–protein interactions leading to autologous catalytic inhibition of *Pf*OPRTase that may regulate flux through this enzyme. The crystal structure shows that active-site residues in this arrangement cannot interact with the substrate to catalyse the reaction. Inhibition through this interaction has not been previously observed or studied in OPRTase and therefore these predictions are under active biochemical investigation in our laboratory.

The N-terminus of OPRTase is longer in the *Plasmodium* genus than in other species and this extension is thought to interact with OMP decarboxylase, the next enzyme in the pyrimidine-biosynthesis pathway (Krungkrai *et al.*, 2004 ▶). The parasite-specific residues in the longer N-terminus form an amphipathic helix α1, the hydrophobic surface of which is sandwiched between α2 and α3 on one side of the *Pf*OPRTase core, while the other predominantly polar surface is solvent-exposed. LCR1, a parasite-specific low-complexity sequence that was deleted in the construct that was crystallized, would have juxtaposed exactly between α1 and α2, which now have a short loop separating them. From this position between α1 and α2, LCR1 would not be expected to interact at the active site nor at the dimeric interface. There is another disordered region of 18 amino acids in length between the antiparallel β5 and β6. This parasite-specific stretch is comprised of an Asp- and Lys-rich low-entropy insert (^182^DDKDILNLKKKTKN­NQDE^199^). While partially conserved in the *Plasmodium* genus, it is absent in other organisms, including humans.

## 4. Discussion   

Nucleotide biosynthesis is an important target in proliferating malarial parasites inside human erythrocytes (Rathod, 2000 ▶, 2001 ▶). In principle, the complete dependence of *Plasmodium* parasites on pyrimidine biosynthesis, combined with the ability of the host, but not the parasite, to salvage preformed pyrimidines, presents many potential opportunities to attack this important human pathogen with selectivity. In one approach, substrate analogues are selectively converted by the pathogen into potent toxins: 5-fluoroorotate (5FO) kills the parasite with low-nanomolar potency and with more than 1000-fold selectivity (Rathod *et al.*, 1989 ▶). A key step in this action is the conversion of 5FO to 5FOMP and ultimately other toxic fluorinated nucleotides by *Pf*OPRTase (Rathod & Reyes, 1983 ▶; Rathod *et al.*, 1992 ▶). Exploiting the structural information on the *Pf*OPRTase enzyme and its differences from the human enzyme may help in the design of even more specific and potent substrate analogues directed at malaria parasites. In a very different approach, high-throughput screening against *P. falciparum* dihydroorotate dehydro­genase and lead optimization of an active triazolopyrimidine has led to potent selective inhibitors of malaria parasites that are in human testing (Baldwin *et al.*, 2005 ▶; Phillips *et al.*, 2008 ▶; Coteron *et al.*, 2011 ▶; Deng *et al.*, 2014 ▶; Gujjar *et al.*, 2011 ▶; http://www.mmv.org/research-development/rd-portfolio).

Among all the early enzymes of the pyrimidine-biosynthesis pathway in *Plasmodium*, the structure of *Pf*OPRTase is the only one that has been unavailable until now. Our first accomplishment here was to obtain crystallizable *Pf*OPRTase. The *Plasmodium* proteome has an abundance of proteins with interspersed low-complexity regions (LCRs), which are often inserted within the coding regions of enzymes (Gardner *et al.*, 2002 ▶; Aravind *et al.*, 2003 ▶). Some AT-rich micro-satellites in the genome manifest themselves in the proteome in the form of continuous stretches of asparagines. These asparagine-repeat stretches are 37 residues in length on average (Zilversmit *et al.*, 2010 ▶). While the evolutionary and functional significance of these LCRs to the parasite is under active debate (Guler *et al.*, 2013 ▶; Muralidharan *et al.*, 2012 ▶; Aravind *et al.*, 2003 ▶), there is agreement that they can hinder overexpression in heterologous systems.

High protein expression was achieved by the removal of a 22-amino-acid asparagine-rich LCR that is close to the N-terminus of *Pf*OPRTase without compromising the catalytic activity of the enzyme. This altered construct was able to complement wild-type OPRTase in the *E. coli* knockout strain JW3617(DE3) and permit its growth in M9 minimal medium. For crystallography, removing dispensable LCRs appeared to reduce conformational heterogeneity since it was not possible to identify a crystallization condition for wild-type *Pf*OPRTase. However, the trimmed *Pf*OPRTase 1–218(Δ37–58) could be crystallized under a number of conditions. The successful removal of this particular LCR region without affecting the catalytic function of the protein can now be rationalized based on the available new structure, since the deleted segment (37–58) would normally be present between α1 and α2, away from the core of the protein.

In the future, it may be possible to find selective active-site binding of inhibitors to this enzyme because the active site of *Pf*OPRTase has a number of amino acids that differ in the parasite enzyme compared with the human enzyme. Remarkably, of the 11 active-site residues at the core of the human enzyme, five are different in the malarial enzyme. Perhaps even more significantly, all five of the unique amino acids are on the back wall of the active site where OMP and PRPP bind, while the fold that wraps around the substrate is well conserved. Additional structures of *Pf*OPRTase 1–218(Δ37–58)-H6 bound to substrates, products and inhibitors should be helpful in further exploiting this.

Beyond the active sites of individual dimers, there are higher order superstructures of *Pf*OPRTase with potential implications for malaria biology and drug design. *Pf*OPRTase 1–218(Δ37–58)-H6, in addition to forming well known dimers at low concentration, seems to form inhibitory multimers at very high concentrations. Inside the unit cell of our *Pf*OPRTase 1–218(Δ37–58)-H6 crystal (PDB entry 4fym) there are 32 polypeptides. Equating this with the unit-cell volume, the enzyme concentration in the crystal is about 18 m*M*, similar to that used to set up the crystals. If these concentrations are approachable in cells, the interactions that are described could be of physiological importance in malaria biology. These protein-level auto-regulatory feedback loops have not previously been recognized. They may work hand in hand with the ability of malaria enzymes to form autologous complexes with nucleic acids (Zhang & Rathod, 2002 ▶).

The short peptide structures involved in interdimer interactions in *Pf*OPRTase reveal novel protein–protein inter­actions that could serve as a template for other more rigid inhibitors directed at the *Pf*OPRTase active site. The loop peptide entering the *Pf*OPRTase active site not only precludes the normal *Pf*OPRTases active-site hood from closing, but the hood domain becomes structured when the β-hairpin of the inhibitory dimer inserts itself over the active site. Since β-hairpins form stable secondary structures in isolation (Blanco *et al.*, 1994 ▶), their analogues may be used to specifically inhibit the enzyme activity of *Pf*OPRTase.

## 5. Summary   

*Pf*OPRTase, an important enzyme in *de novo* pyrimidine biosynthesis in malaria parasites, has been overexpressed and crystallized. This is an important advancement in our quest to develop many different and increasingly better inhibitors to block malaria pyrimidine biosynthesis with selectivity. The present structure will help in the development of nucleotide precursors as prodrugs against parasites and also inhibitors that directly block OPRTase activity in malaria parasites. As an additional bonus, the new structure hints at a unique mechanism for autoregulation of *Pf*OPRTase activity in the parasite based on interdimer protein–protein inter­actions. The nature of this interdimer structure points to a natural lead peptide inhibitor that could also guide future structure-inspired antimalarial drug-development strategies.

## Supplementary Material

PDB reference: orotate phosphoribosyl­transferase, 4fym

Supporting Information.. DOI: 10.1107/S2053230X1500549X/hv5289sup1.pdf

## Figures and Tables

**Table 1:** Data-collection and refinement statistics

Data collection	
Diffraction source	Beamline 23-ID-B, APS
Wavelength ()	1.09
Temperature (K)	100
Detector	MAR Mosaic 300 mm CCD
Crystal-to-detector distance (mm)	320
Rotation range per image ()	0.5
Space group	*P*2_1_2_1_2_1_
*a*, *b*, *c* ()	114.77, 152.49, 167.76
, , ()	90, 90, 90
Resolution range ()	50.02.6 (2.632.60)
Total No. of reflections	1819537
No. of unique reflections	90605 (2832)
Completeness (%)	98.9 (93.9)
Multiplicity	5.6 (4.6)
*I*/(*I*)	22.6 (1.97)
*R*_r.i.m._ †	0.081 (0.711)
*R*_merge_	0.073 (0.629)
Overall *B* factor from Wilson plot (^2^)	51.16
Refinement statistics	
Resolution range ()	40.472.6 (2.662.59)
Completeness (%)	98.5
No. of reflections, working set	85965 (5765)
No. of reflections, test set	4588 (311)
Final *R*_cryst_	0.197 (0.312)
Final *R*_free_	0.218 (0.345)
No. of non-H atoms	
Protein	14453
Ion	165
Water	264
Total	14882
R.m.s. deviations	
Bonds ()	0.016
Angles ()	1.839
Average *B* factors (^2^)	
Protein	62.7
Ion	74.1
Water	47
Clashscore	3.81
Ramachandran plot	
Favored regions (%)	98.8
Additionally allowed (%)	1.1
Outliers (%)	0.1

† *R*_r.i.m._ = *R*_merge_[*N*/(*N* 1)]^1/2^, where *N* is the data multiplicity.
