# Supplementary material for: Structure of Plasmodium falciparum orotate phosphoribosyltransferase with autologous inhibitory protein–protein interactions
Source: Acta Crystallogr F Struct Biol Commun. 2015 Apr 21;71(Pt 5):600–8. doi: 10.1107/S2053230X1500549X (PMC4427171; doi:10.1107/S2053230X1500549X)
Supplement: Supplementary file 1 [file f-71-00600-sup1.pdf]

STRUCTURAL BIOLOGY COMMUNICATIONS

Volume 71 (2015)

Supporting information for article:

Structure of *Plasmodium falciparum* orotate phosphoribosyltransferase with autologous inhibitory protein–protein interactions

Shiva Kumar, Kalyanaraman Krishnamoorthy, Devaraja G. Mudeppa and Pradipsinh K. Rathod

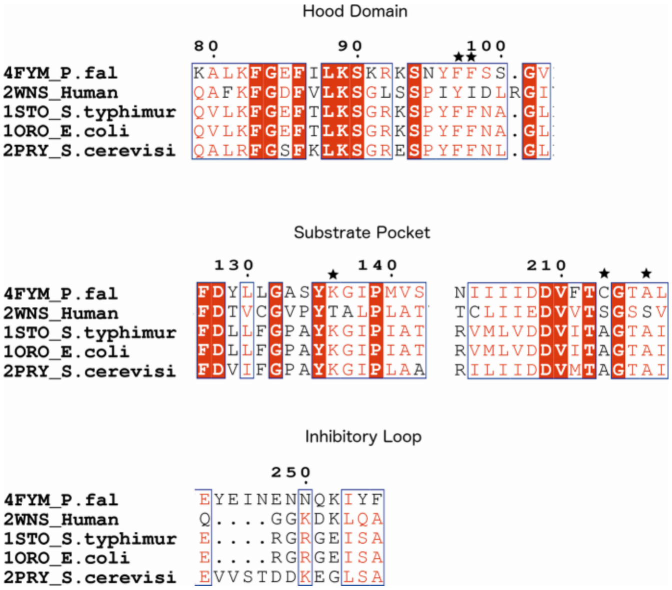

Figure S1 Multiple sequence alignment of OPRTase homologues with available structures. Sequence similarity around the hood domain, substrate binding pocket and inhibitory loop is depicted in separate boxes. PDB id and the organism names are shown for each sequence. Residues are numbered according to the *Plasmodium falciparum* sequence. Conserved residues are shown with red background, while the semi-conserved residues are shown in red fonts in white background. Residues that are expected to make contacts with the ligand, based on the human structure (PDB id: 2WNS), are denoted with a black star.

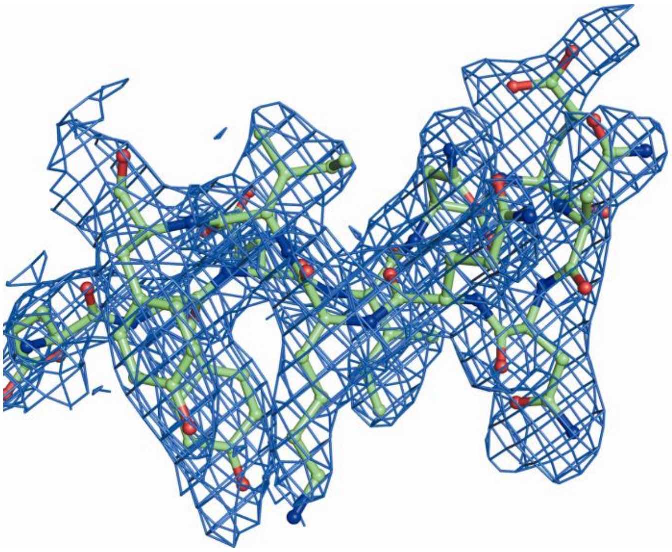

Figure S2 Simulated annealing omit map of the *P. falciparum* OPRTase inhibitory loop. Blue mesh depicts the 2fo-fc map calculated using the simulated annealing omit-map procedure and contoured to 1σ. The entire inhibitory loop (^{243}EYEINENNQKIY^{254}) from all the eight chains were omitted for the calculation. Quality of the map shows that the inhibitory loop is modeled entirely within an unbiased electron-density.
